# 293 Risk of Clostridium Difficile Enterocolitis in SJS/TENS Patients Treated with Antibiotics

**DOI:** 10.1093/jbcr/irad045.268

**Published:** 2023-08-29

**Authors:** Savannah Skidmore, Ashley Hink, Steven A Kahn, Deepak Ozhathil

**Affiliations:** Medical University of South Carolina, Charleston, South Carolina; Medical University of South Carolina, Charleston, South Carolina; The South Carolina Burn Center/Medical University of South Carolina, Charleston, South Carolina; South Carolina Burn Center at MUSC, Charleston, South Carolina

## Abstract

**Introduction:**

Stevens-Johnson Syndrome (SJS) and Toxic Epidermal Necrolysis Syndrome (TENS) are both rare conditions derived from adverse drug reactions. SJS/TENS patients are often treated with antibiotics, both prophylactically and therapeutically, for secondary infections. However, the subsequent development of *Clostridium Difficile (C. Difficile*) enterocolitis is unknown. Due to the rarity of SJS/TENS, institution trials are limited in their data quantify the additional risk of sequelae such as enterocolitis development after antibiotic administration. Therefore, we used a large commercially available database derived from inpatient hospital coding data to assess the incidence of *C. Difficile* enterocolitis within 2 weeks of antibiotic administration after development of SJS/TENS.

**Methods:**

: Our study utilized a large commercially available database. Inclusion criteria identified adult patients ( >18 years old) diagnosed with SJS/TENS between 2002 and 2022, who were treated with antibiotics within 2 weeks of diagnosis, and who subsequently went on to develop *Clostridium Difficile* enterocolitis within 6 months. We compared our cohort to a control group which did not receive antibiotics and propensity score matched them for age, sex, race, HIV status, cancer, transplant status, and severe sepsis and SIRS and analyzed the risk of developing *C. Difficile* enterocolitis.

**Results:**

There were 4,792 SJS/TENS patients who did not receive antibiotics and 5,652 who did. Our matched cohorts contained 4,595 patients. The relative risk of mortality between our cohorts was 4.962 (RR: 2.562, CI 95% 2.125-3.088; p< 0.0001). SJS/TENS who received antibiotics developed *C. Difficile* 68/4595 vs 30/495 and had a relative risk of 0.827% (RR: 2.267, 1.478-3.476, CI 95%; p< 0.0001). Therefore, an SJS/TENS patient incurred a 2.267 times higher risk of developing *C. difficile* enterocolitis after receiving antibiotics.

**Conclusions:**

While causality cannot be affirmed with retrospective data, our findings do suggest that patients with SJS/TENS due to the statistically significant risk of *C. Difficile* enterocolitis development. However, the overall absolute risk of *C. difficile* enterocolitis remains disproportionately low in this population that warrants further exploration.

**Applicability of Research to Practice:**

SJS/TENS patients are difficult to develop evidence-based treatment plans for because of their rarity and the multitude of conflicting small cohort studies available in the literature. Our study utilizes a large database to more robustly suggest that clinicians should be cautious about administering antibiotics to patients diagnosed with SJS/TENS.

